# Ten Simple Rules for Running Interactive Workshops

**DOI:** 10.1371/journal.pcbi.1003485

**Published:** 2014-02-27

**Authors:** Katrina Pavelin, Sangya Pundir, Jennifer A. Cham

**Affiliations:** European Bioinformatics Institute (EMBL-EBI), European Molecular Biology Laboratory, Wellcome Trust Genome Campus, Hinxton, Cambridge, United Kingdom; University of California San Diego, United States of America

## Introduction

Do you have a difficult problem to solve? Are you writing a grant proposal involving several stakeholders? Do you want to gather user feedback on a resource, tool, or service? Or perhaps you need to improve a process or way of working in a team? To address problems such as these, we recommend holding an interactive workshop. We find that the dynamic nature of such workshops encourages creative thought and can quickly yield ideas and solutions [1].

**Figure 1:**
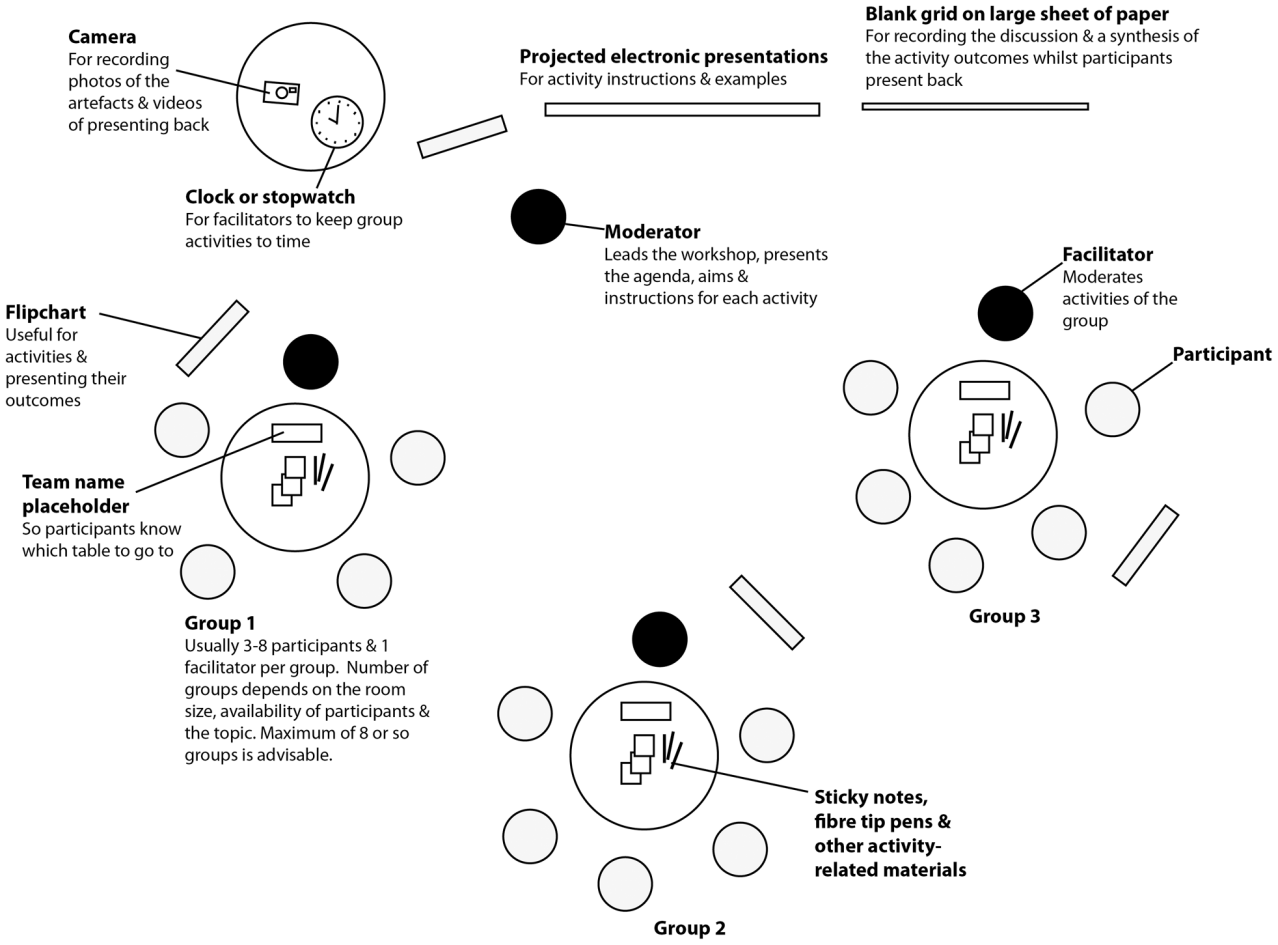
Example room layout for an interactive workshop. This bird's-eye view shows the setup for supporting group-based, facilitated activities around a specific topic, problem, or project. The moderator oversees the workshop with the help of facilitators, who are briefed in the aims and methods of the activities. Alternatives include “circuit training” layout, where each table is an activity station and the participants move around the room.

Our ten simple rules aim to empower you to design and lead your own successful interactive workshops. We define an “interactive workshop” as a structured set of facilitated activities for groups of participants who work *together* to explore a problem and its solutions, over a specific period of time, in one location. Participants can be users, potential users, team members, customers, or stakeholders. Figure 1 shows a typical layout; note if you have five or fewer participants, consider having just one group.

An interactive workshop is distinct from a standard meeting because it aims to stimulate creativity through collaborative working. A meeting, in contrast, usually involves planning and reporting work with attendees sharing their individual points; it does not involve group activities “live” in the meeting. Also, meetings may be an hour or less, whereas the minimum time needed for an interactive workshop is 2–3 hours.

We also do not include training workshops, talks, or seminars in our definition, because from our experience they tend to comprise lectures and tutorials, not brainstorming activities. However, it could be possible to have an interactive workshop session as part of a longer training course.

## Rule 1: Decide Whether an Interactive Workshop Is the Right Choice

Interactive workshops can be useful in many situations, with both internal and external participants; see [2] for an example case study. Interactive workshops may be suitable for:

- gathering ideas for research grant proposals;
- ascertaining user requirements for bioinformatics services;
- generating ideas for designing web/software interfaces;
- solving problems, such as process improvement or work strategies;
- deciding priorities, strategy, and vision;
- improving working relationships through team building, such as part of retreats.

Before you start planning, it is important to determine whether an interactive workshop is the right choice. For instance, they are not usually advisable at the inception of a project when you need to identify the goals. Organisation leadership, policy, and many other factors may determine this. However, once objectives have been identified and agreed upon, then an interactive workshop can be valuable to explore *how* to meet them.

Interactive workshops are also unsuitable when you have firm alternatives to evaluate (like mock-ups for a website). It would be better to get individual feedback and then collate the results.

Another consideration is that interactive workshops can require extra time and resources to plan and deliver because activities, templates, and materials need to be prepared in advance and more people may be required for facilitating the activities. We suggest if there are significant constraints, especially short timescales, it may be more appropriate to hold a standard-format meeting [3].

## Rule 2: Choose Participants Carefully

Descriptions of your target groups (or “user profiles” [4]) may help guide your choice of participants for an interactive workshop. Aim for diversity in experience, opinions, seniority, and interests. For external participants, send out an electronic “screener” survey to find out if they truly represent your target profiles and use this information to assign groups for the activities. You may wish to split colleagues into different groups because separating people who usually work together exposes them to alternative perspectives and new thinking, thus stimulating creativity.

If you are not able to choose the participants yourself, you will still need to find out who they are and see how their perspective and/or background fits with the objective(s) of the workshop. Consequently, you may need to factor this into your analysis of the outcomes.

## Rule 3: Identify Suitable Activities

Before you start planning, think about how you (as the person managing the delivery of the interactive workshop) will present the outcomes in a talk or report. Consider: What are the tangible aims of the workshop? What specific information do you need to capture? Tailor the activities to these specific goals. Where possible, use engaging activities, such as “game-storming” techniques [5], to motivate your participants. By using visual metaphors and games you may further encourage creative thought, as this allows the rules of everyday life to be suspended for exploring problems in new, sometimes unorthodox, ways.

Some participants may be sceptical about the value of “just playing games.” However, once you clearly explain the aim of each activity and how it is tailored to solve the problem, most participants will engage positively. For a checklist of how to introduce an activity to a group, see [6].

## Rule 4: Identify Facilitators and Brief Them

Facilitators coordinate and assist group discussions and activities during an interactive workshop. You will need to carefully brief these helpers to ensure they know how to moderate and are familiar with the aims and practicalities of the activities that are scheduled. You may also wish to have an overseeing moderator who presents the aims, agenda, and activity instructions (Figure 1).

Most importantly, you should emphasise that facilitators need to be impartial coordinators: neither contributing ideas, nor evaluating them, but rather encouraging input from participants in their group. They keep discussions on time, and remind participants to note down all their points, sometimes actually doing this for them whilst they are speaking. Facilitators may also note further ideas onto the artefacts as they arise during the presenting-back phase and subsequent discussion with everyone in the room.

It is not essential for facilitators to have substantial domain expertise, but it may be helpful for them to have at least a basic understanding of the concepts being explored. For example, for activities to prioritise items it helps if facilitators can clarify what the items are if participants have questions, or give specific examples as illustrations if needed.

On the day of the workshop, the facilitators (or moderator) will monitor the groups and, if there is time, they may consider reshuffling them during the breaks, as this can boost creativity.

## Rule 5: Consider Logistics, Facilities, and How to Record Outcomes

In advance, arrange to view the room that you will be using for the interactive workshop. The physical space(s) and equipment will influence the activities you run. Find out:

- Are there flip charts or blank walls for recording your participants' ideas? If not, do you need to order equipment such as foam boards?
- Can you rearrange the tables into small groups, or are there additional rooms available?
- Is there audio-visual equipment, such as microphones for giving activity instructions?
- Is there a projector for electronic presentations of the activity instructions and templates?
- Are there areas for circulating during breaks?
- Can you arrange refreshments and/or catering?

**Figure 2:**
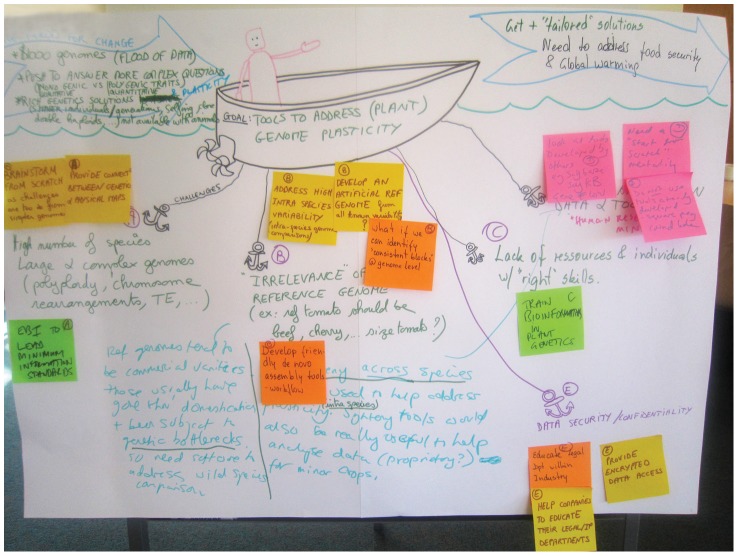
Example of a workshop artefact: The output of the “Speed Boat” activity. The aim of this activity is to identify improvements that need to be made, for instance, to a product or service. The boat and anchors are drawn on paper as a template before the workshop. During the activity, the groups add their ideas in pen: they write the goal of the workshop on the boat and the challenges to achieving this goal by the anchors. We also include “positive forces for change”—things that are moving the project towards the goal—as “wind arrows” flanking the boat. The sticky notes have been added after the activity by the facilitator during the presenting-back stage and group discussion. The sticky notes have been labelled with the letters A to E for reference; note that a labelling scheme may be helpful for the analysis and report. This activity was adapted from p. 206 in [5]; also watch this video for more hints: http://www.youtube.com/watch?v=xwVbcioYvdM.

The groups' outputs are called “artefacts” (see Figure 2 and http://www.youtube.com/watch?v=xwVbcioYvdM for an example). Artefacts could be whiteboards, flip-chart paper sheets, drawings and/or sketches, canvasses (such as for the “canvas sort” activity in [2]), or 3D objects (such as boxes, as in the “design the box” activity in [5]). It is wise to take photographs of all artefacts on the day; we have found that paper is easily lost! Recording video summaries of the groups presenting back may also help clarify the information captured. Note that you will need to obtain consent from participants for video footage or photographs.

From our experience, interactive workshops are not suitable for remote participation, such as via webcasting, because the collaborative aspects of the group work cannot be easily shared remotely. If input from participants in distinct locations is necessary, consider running multiple smaller interactive workshops in different places.

## Rule 6: Plan the Agenda

You will need a minimum of 2–3 hours to run an interactive workshop but, ideally, a full day. For external participants, also consider the impact of travel arrangements on timing.

**Figure 3:**
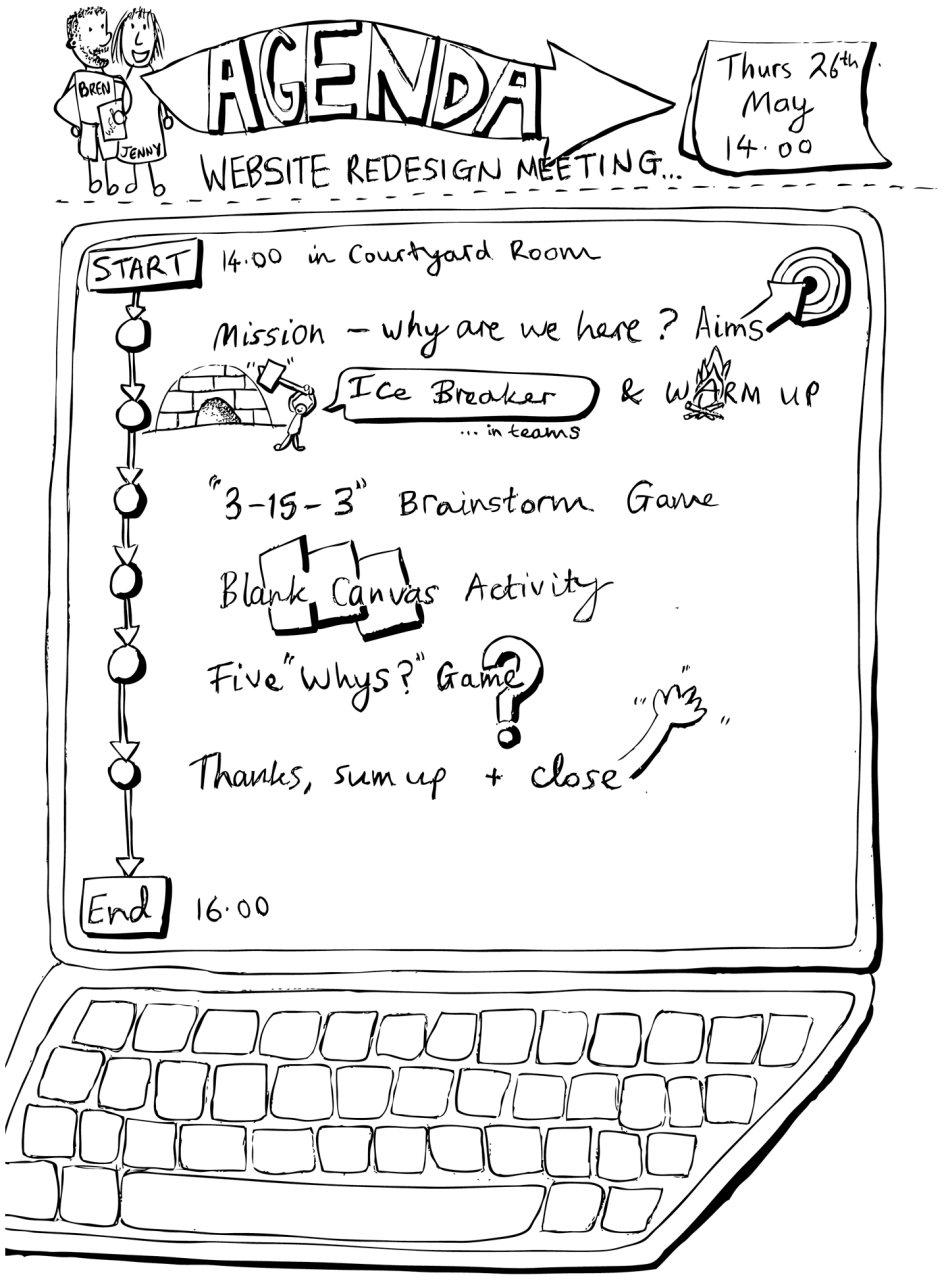
Example of a visual agenda. This was created for an internal workshop held to gather ideas for a website redesign process. Visual agendas are useful for setting the creative tone needed for successful interactive workshops.

Ensure the agenda balances different types of activities, such as individual, paired, and collective tasks. Start with a hands-on activity as soon as possible and keep electronic-based presentations to a minimum—these can sap the creative atmosphere. Bear in mind the phases of a creative workshop: “opening” (generating ideas), “exploring” (experimenting with the ideas, finding patterns), and “closing” (evaluating, deciding, and listing actions) [5]. To conclude each activity, ensure you schedule enough time for presenting back to the group and discussing outcomes of each activity. This shows participants that their feedback and participation matters, and it provides an opportunity for clarification. Where possible, we use visual agendas because they set the participants' expectations of the day and prepare them for the interactive nature of the workshop (Figure 3).

Interactive workshops can be demanding. To maintain energy levels, plan breaks, provide refreshments, and aim to finish early. You may need to schedule the high priority activities earlier in the day in case enthusiasm wanes as you proceed. Alternatively, if you have participants who have travelled long distances and/or from different time zones, schedule the most important activity for later in the day.

Remember: planning is important, but you will need to be flexible with the timing on the day, for instance, if an activity is completed early or it overruns.

## Rule 7: Market Your Interactive Workshop As a Networking Opportunity

Networking is a great way to incentivise participation in a workshop with external candidates. Send named invitations and advertise the other organisations that will be present. You may wish to encourage the participants to network after the event by sharing their contact details. This can also be a useful contact list for your own future events. Remember to always get consent for how you intend to use contact information.

To encourage socialising at the interactive workshop, consider using games such as the “Low-tech Social Network” or “Show and Tell” [5]. We also recommend using name badges.

## Rule 8: Get the Best from Your Participants

As an interactive workshop leader, you need to encourage people to work together in a short space of time. We recommend displaying a table plan near the entrance to help participants settle quickly. We also try to give the groups amusing names to help set the creative tone and/or workshop theme.

Get the participants to introduce themselves, for example, by saying where they work, what they do, and why they chose to attend. This can be done for the whole room or group by group if it is a very large workshop. We find it is important for participants to know who is in the room before they will be comfortable sharing their ideas. After this, use “warm-up” and/or “ice-breaker” activities to stimulate collaborative working; for ideas, see [7].

For some activities, you may wish to have an example artefact pre-prepared, perhaps using a “toy” example. This can be more instructive than a list of instructions. Also have a template prepared for recording a synthesis of the presenting back (Figure 1).

Reflective personality types may find “on the spot” thinking uncomfortable [8], so provide information about the workshop aims in advance to help them prepare. On the day, using what we call a “creative silence” approach, where participants brainstorm ideas on sticky notes individually before sharing with the group, can help generate ideas and ensure that everyone participates.

If you have an international audience or diverse disciplines represented at your interactive workshop, you may need to do some background research. For example, some cultures have a tendency to be more reserved, whilst others may “warm up” quickly and may even need “cool down” activities to get the best results.

We find that details get noticed by the participants, so having creatively designed placeholders, good quality refreshments, a tidy and bright room, etc. make for a positive experience. The participants usually appreciate this and will in turn give more energy to taking part in the activities.

## Rule 9: Follow Up with Your Facilitators and Create a Post-Workshop Report

Immediately after the interactive workshop, ask your facilitators for their top three findings on the outcomes, and their main feedback on how the workshop went as a whole. It is best to do this face-to-face, so that you can reach a consensus quickly (because you will all be tired!). At the planning stage, schedule a meeting to do a more comprehensive analysis of the findings the day after the workshop. During the analysis you will need to synthesise and summarise the information on the artefacts.

You will probably need to present these outcomes in a report for whoever requested (and/or funded) the workshop. The executive summary is key because it highlights the main findings. We also suggest including the methods used for each game so that the reader can see how your results were gathered. Include quotes from participants to emphasise the results. Present your findings visually wherever possible—for instance, use graphs, charts, and photographs. Highlight any patterns that you identify and consensus from the delegates (e.g., “80% chose A, 20% chose B”).

## Rule 10: Follow Up with Your Participants

Ask for feedback on the interactive workshop format: this is the best way to learn what works, and it shows your participants that you value their input. It is easiest if you can get the participants to fill out a short survey, ideally in the final coffee break so they complete it before they leave. Alternatively, you could send a thank you note to each participant with a link to a short electronic survey.

If the workshop was with stakeholders in a project (as opposed to users), then you may wish to share a report of the findings and a list of actions. It is important to do this soon after the event to maintain momentum. To strengthen the collaborations made with external participants, we have often arranged talks and outreach events at their institutes. Often the initial workshop gives us an insight into their requirements, which allows us to tailor activities to their needs.

Additionally, you may wish to contact participants for future projects (if you have obtained their consent for this). For instance, we ran a user-experience–based interactive workshop for a European Bioinformatics Institute (EBI) service, and once we had developed our ideas further, we invited the participants back to take part in usability testing of mock-ups of interface designs. We also asked if their colleagues would like to take part. This led to a day of testing with new participants and resulted in new collaborations. Networking with the participants is a good way to find opportunities for outreach, collaboration and future interactive workshops.

**Further Reading**

- Stephenson J, Galloway A (2012) The secrets to workshop success. Free e-book. ISBN: 978-87-403-0174-8. Available: http://bookboon.com/en/the-secrets-to-workshop-success-ebook. Accessed 14 August 2013.
- Sibbet D (2010) Visual Meetings: How graphics, sticky notes and idea mapping can transform group productivity. New Jersey: John Wiley & Sons.
